# A Non-inductive Coil Design Used to Provide High-Frequency and Large Currents

**DOI:** 10.3390/s24072027

**Published:** 2024-03-22

**Authors:** Qing Zhu, Yu Su

**Affiliations:** School of Mechatronic Engineering, Xi’an Technological University, Xi’an 710021, China; zhuqing@st.xatu.edu.cn

**Keywords:** non-inductive coil, pulse technology, current source, current pulse

## Abstract

Currently, cutting-edge, high-frequency current sources are limited by switching devices and wire materials, and the output current cannot take into account the demands of a high peak and low rise time at the same time. Based on the output demand of a current source, a non-inductive coil for providing high-frequency, high current sources with low rise times is designed. The coil is appropriately designed according to the principle of the ampere-turn method, where several turns of wire are utilized to linearly synthesize the current to obtain high-frequency currents with amplitudes up to 30 kA. However, the inductance formed after winding the coil could possess a hindering effect on the high-frequency current. In the present investigation, based on the law of energy conservation and utilizing the principle of transformer coupling, the inductor’s hindering effect on high-frequency currents is appropriately eliminated by consuming the stored energy of the inductor innovatively. Theoretical calculations and practical tests show that the inductance of a two-layer 28-turn coil is 42 times smaller than that of a two-layer, 28-turn perfect circular spiral PCB coil. The measured inductance is only 6.69 μH, the output current amplitude is calculated to be up to 33 kA with a rise time of 20 ns, and the output waveform corresponding to a 1 MHz square wave is not remarkably distorted. This effective design idea could be very helpful in solving the problem of high peak values and low rise times in high-frequency, high-current source output design.

## 1. Introduction

High-power pulsed power technology originated in the 1930s from research on generating rays using capacitors, and the first publication of microsecond pulsed X-rays from a high-voltage pulsed power discharge point was made by Kingdon and Tanis in 1930 [1].

Among the pulsed current sources, the adjustable high-frequency, high-current pulse source, as a new type of controllable pulsed current source, plays a central role in the field of parameter calibration of high-precision, high-volume range AC measurement equipment [2]. Additionally, the calibration current source is currently in the stage of continuous improvement [3]. In general, there are two main research directions in the present mainstream electrical energy design research.

The first is how to generate high-frequency, high-current sources with amplitudes exceeding the kilo Ampere class. Kovalchuk et al. [4] described the design and testing of a pulsed source with an adjustable setting which was capable of delivering currents of 400 to 600 kA in a 200 to 800 ns rise time. The complete system reaches the order of tons and has dimensions of 1 × 0.6 × 0.81 m^3^ (with a pumping system). However, the generator equipment was too bulky and complex to be put into practical use. Bazanov et al. [5] created a destructive current source based on the bursting mode with a current amplitude of up to 10 MA and a rise time of 120 ns. This current source is disposable and the current waveform is not controllable. In addition, magnetic pulse compression (MPC) systems have been designed to achieve a rise time of 307 ns and a peak power of 30.2 MW [6], but the waveforms are reproducible only up to 7 kHz.

The second one is how to control the accuracy of the output waveform and generate the waveform without distortion. Carvalhaes-Dias et al. [7] designed a proportional to absolute temperature (PTAT) current source with a maximum nonlinearity of 0.44 ppm/C. Wei et al. [8] designed a current source with a ripple ratio of 0.1% and an amplitude of 100 A based on the requirement of Hall element detection. The measurement objects were almost sinusoidal, representing the waveform of the other side of the waveform reduction without further discussion.

It can be found that the world’s top high-frequency current source pulse design research cannot simultaneously meet the output current of kiloamps and the rise time of tens of nanoseconds.; this requires new design ideas and designs to meet the special requirements of the amplitude of this large, short rise.

In this paper, based on the law of energy conservation and utilizing the principle of transformer coupling, an innovative approach is proposed to eliminate the hindering effect of inductors on high-frequency currents by depleting the stored energy of inductors. To this end, a non-inductive coil for outputting high-frequency, high-current sources with low rise times is designed based on the output design requirements of a current source. According to the principle of the ampere-turn method, the coil uses several turns of wires connected in parallel to linearly synthesize multiple small currents to arrive at a high-frequency current with an amplitude of up to 30 kA. However, the parasitic inductance of the coil could hinder the high-frequency current with an amplitude of up to 30 kA, which is mainly reflected in the delayed rise time and waveform distortion.

## 2. Non-Inductive Coil Design Preparation

In order to meet the output requirements of a high-frequency, high-current, single-cycle pulse source made by a Chinese laboratory, the other electromagnetic induction equipment in the laboratory were tested for extreme parameters.The output limit design requirements for this current source are presented in Table 1.

Since the current source of the output current amplitude and rise time requirements are extremely high, the current conventional current output program cannot meet the requirements. It is mainly reflected in the current mature commercial switching devices, which can be achieved within 25 ns switching speed products. Actually, the switching current amplitude indicators are not more than 100 A, which is more than a hundred times the gap with the design of 30 kA and cannot be utilized to increase the amplitude of the current simply by paralleling the switching device approach. Here, we propose a new output design approach to meet the design specifications.

### 2.1. Development of a Theoretical Model for Eliminating Inductive Effects

Consider first how the current amplitude target of the current source is reached. According to the magnetic circuit Ohm’s law and magnetic circuit Kirchhoff’s law [9],

(1) $$\begin{array}{c} \underset{j}{\sum }N_{j}I_{j}=\underset{j}{\sum }R_{j}\Phi_{j}\; \end{array}$$

where *N_j_* represents the *j*-th coil turn of the coil, *I_j_* denotes the current flowing through the turn, *R_j_* is the magnetic resistance on the *j*-th coil turn, and $\Phi_{j}$ represents the changing magnetic flux passing through the *j*-th coil.

This formula shows that the algebraic sum of the number of ampere-turns around a closed path in a magnetic circuit is equal to the algebraic sum of the magnetoresistance and the flux magnitudes. Therefore, the magnetic field effect of a single turn of high current can be simulated by superposing the magnetic path of multiple turns of a small current. Let the total target current be *I*_0_, the current passing through a single wire be *I_1_*, and the number of turns of the winding coil be represented by *N*. The corresponding formula reads as follows [9]:

(2) $$\begin{array}{c} I_{0}=NI_{1} \end{array}$$

**Figure 1:**
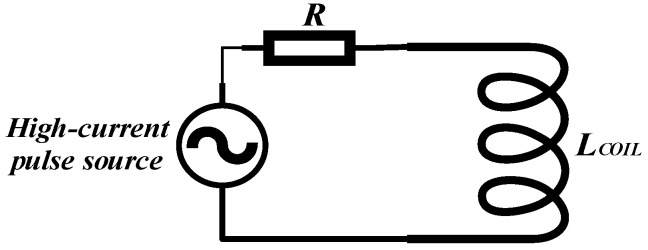
High-frequency current equivalent circuit of the coil.

This is the basic principle of the ampere-turn approach, where the algebraic sum of the current per turn is equal to the algebraic sum of the product of the magnetoresistance and the magnetic flux. By winding a wire into a coil and treating one side of the coil as a whole, a large current pulse several times the current flowing through a single wire is obtained. The equivalent circuit is schematically presented in Figure 1.

In Figure 1, *R* denotes the resistance of the coil and *L* represents the equivalent inductance of the coil. This paper focuses on the hindering effect of the coil’s equivalent inductance on high-frequency currents. The mathematical formulation of the inductance can be introduced by Maxwell’s equations:

(3) $$\begin{array}{c} v\left(t\right)=\frac{d\Phi }{dt}=\frac{dLi\left(t\right)}{dt}=L\frac{di\left(t\right)}{dt}\; \end{array}$$

**Figure 2:**
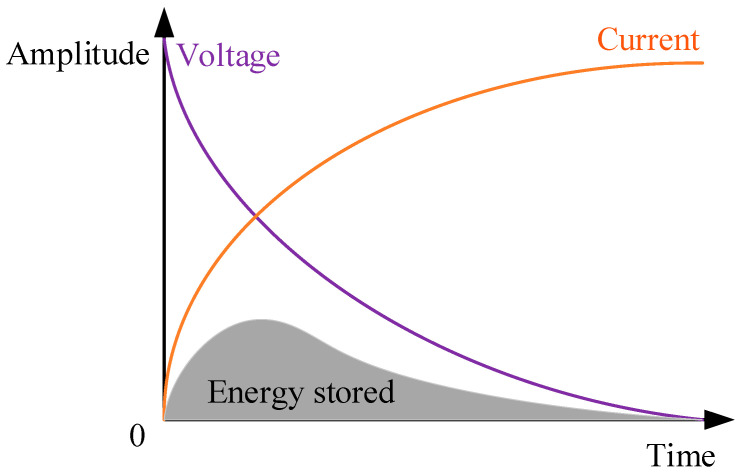
Graphical representation of the voltage, current, and energy of an inductor as a function of time [10].

Equation (3) gives the essential reason why the inductor hinders high-frequency current: high-frequency current causes drastic changes in the electromagnetic field, and part of the energy of the current is stored by the magnetic field and is released when the current becomes stable, causing obstruction. Therefore, it can be considered that the storage and release of inductive energy is the essence of the inductor on the high-frequency current obstruction effect. This plays a major negative impact on the work of the high-frequency circuit but is also the main factor of the high-frequency current output waveform distortion, reflected in the hysteresis phenomenon of the rising edge of the square-wave pulse, as illustrated in Figure 2 [9,10].

Therefore, it can be assumed that to achieve the design requirement of tens of nanoseconds of rise time, the hindering effect of the equivalent inductance of the output coil on the high-frequency current must be eliminated.

In this paper, from the perspective of energy conservation, the structural design of the magnetic field changes with the loss of energy in advance of the conversion and consumption, so that the energy stored in the inductor is reduced or even not, so as to weaken or even eliminate the obstruction effect of the inductor on the high-frequency current.

The mutual inductance of two coils has the following relation [10]:

(4) $$\begin{array}{c} M=k\sqrt{l_{1}l_{2}}\; \end{array}$$

where *M* denotes the mutual inductance and k signifies the coupling coefficient, whereas *l*_1_ and *l*_2_ represent the self-inductance of the two coils. Assuming that this hollow transformer is ideal, i.e., the element is a full coupler and the two coils are identical, *k* = 1 and *l*_1_ = *l*_2_.

For the primary end of the connected circuit, the induced voltage of the mutual inductance is opposite to that of the self-inductance. At this stage, it can be assumed that the inductance of the coil at the primary end is practically eliminated and only the dynamic resistance impedance due to transformer coupling needs to be taken into account, thus virtually eliminating the effect of the coil inductance on the dynamic circuit. In essence, the energy stored in the inductor to impede changes in the electromagnetic field will be transformed and consumed in the secondary coil according to the transformer coupling effect, thus eliminating the impedance of the inductor on the high-frequency signal in the engineering sense.

According to the impedance calculation formula of the transformer [10],

(5) $$\begin{array}{c} \;Z_{1}=n^{2}Z_{2} \end{array}$$

Considering that the secondary coil and primary coil are completely consistent at this time, the value of n is obviously equal to one, and thereby

(6) $$\begin{array}{c} Z_{1}=Z_{2} \end{array}$$

**Figure 3:**
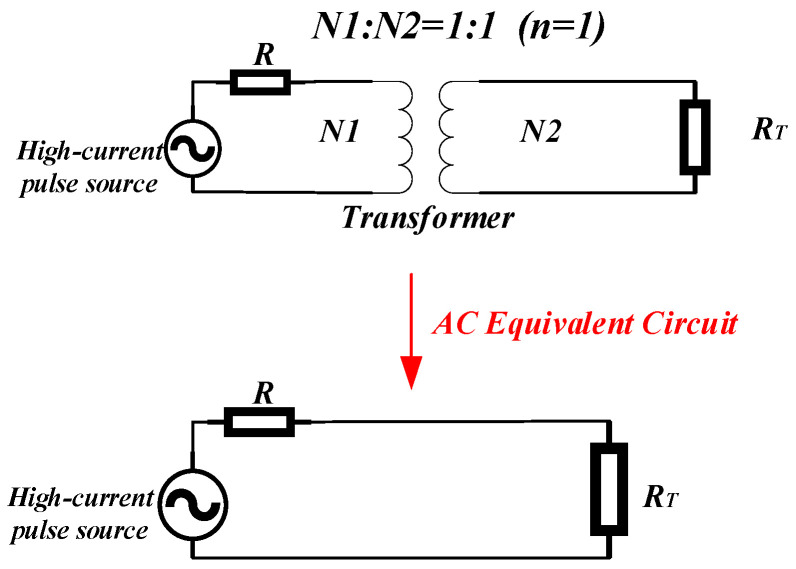
Equivalent diagram of a high-frequency circuit for canceling inductance.

When *Z*_2_ is unloaded, there is only the impedance of the secondary coil at this point. Clearly, the resistance, RT, on the secondary coil is only the resistance generated by the wire. Its high-frequency circuit schematic representation is shown in Figure 3.

**Figure 4:**
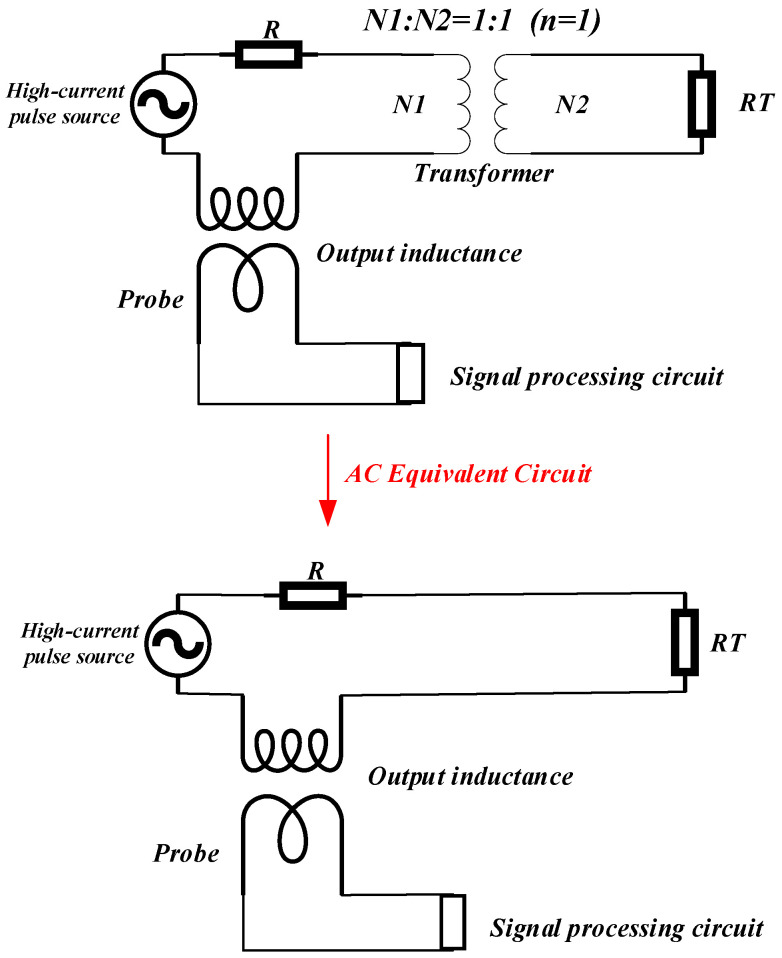
High-frequency circuit equivalent diagram of a non-inductive coil.

However, taking into account the measurement requirements, once the coil is fully coupled, the magnetic energy that is theoretically completely recycled cannot produce magnetic field changes, and also cannot output electromagnetic signals, which as an output coil loses its design significance. Therefore, it cannot completely offset the coil’s own inductance and the primary end of the inductance is not completely eliminated as a test point. The final equivalent circuit of the non-inductive coil is illustrated in Figure 4.

### 2.2. Coil Electromagnetic Parameter Design Model

For a coil utilized to output high-frequency current, there are three core electromagnetic parameters: resistance, inductance, and capacitance. The modeling of these three parameters is in turn discussed in the following.

#### 2.2.1. A Model for Calculating the Coil Resistance

Obviously, the resistance on the coil is simply the resistance generated by the wire. At this point, it can be derived directly using the resistance definition formula. Therefore, the formula for the coil resistance is as follows:

(7) $$\begin{array}{c} R=\frac{\rho l_{length}}{wh_{L}} \end{array}$$

where *ρ* denotes the resistivity of the wire, *l_length_* represents the length of the wire, *w* is the width of the copper-clad wire, and *h_L_* is the thickness of the copper-clad wire.

#### 2.2.2. A Model for Calculating the Coil Inductance

For the unmodified PCB helical coil, the approximate formula of inductance presented in Equation (8) is given according to the work of Zhao [11]. By establishing electromagnetic field simulation models of spiral coils with various shapes, Zhao [11] utilized the electromagnetic model and the principle of electromagnetic induction to give several approximate mutual inductance formulas between spiral PCB coils. Through establishing spiral coils with specified shapes, the PCB coil measured its mutual inductance and verified the validity of the fitting formula. Under certain conditions, the formula can be approximated as the spiral tube mutual inductance calculation formula, verifying the theoretical value and physical meaning of the formula.

(8) $$\begin{array}{c} L_{0}=\left[\frac{\mu_{0}N^{2}D_{AVG}C_{1}}{2}\right]\left.[\ln\left(\frac{C_{2}}{p}\right)+C_{3}p+C_{4}p^{2}\right)],\; \end{array}$$

where $\mu_{0}$ is the vacuum permeability, *N* is the number of turns, and $D_{AVG}$ denotes the arithmetic mean of the inner and outer diameters. The factors *C*_1_–*C*_4_ represent the fitting coefficients, which are determined by the outer shape of the coil. In the above relation, *p* is the filling ratio, which is given by

(9) $$\begin{array}{c} p=\left(D_{OUT}-D_{IN}\right)/\left(D_{OUT}+D_{IN}\right) \end{array}$$

At this point, the coil is employed as the primary coil of the transformer, the corresponding secondary coil is added, and the secondary coil is shortened to have no load. According to the theoretical analysis above, the inductance at this point will be completely converted into the static impedance of the secondary coil. However, this is the assumption that the secondary coil and the primary coil are fully coupled. In fact, if the primary coil as part of the measurement window is not coupled with the secondary coil, then we only need to calculate the uncoupled part of the inductance and the uncoupled part of the reduced impedance can be brought about by the impact. The part, for ease of calculation, can be regarded as a straight wire generated inductance.

Zhong [12] derived an approximate formula for the inductance of a single wire on a PCB based on the definition of inductance and the principle of electromagnetic induction:

(10) $$\begin{array}{c} L_{line}=2l\times \left(\ln\left(\frac{2l}{w}\right)+0.2235\frac{w}{l}\right)\times 10^{-9} \end{array}$$

The derivation of Equation (10) is based on the fact that the thickness of the PCB wire is much smaller than the width and length of the wire and can be regarded as an ideal conductive flat plate. Based on the relevant knowledge of electrodynamics, the electromagnetic field model of the conductive flat plate was established, and the magnetic field energy storage was calculated based on the magnetic field model. Since the essence of inductance is the storage and release effect of magnetic field energy, the calculation formula of inductance can be derived from magnetic field energy storage.

The mutual inductance between wires with the same current direction is equal to the product of the mutual inductance coefficient between the two wires and the sum of the inductances of the two wires. However, there are so many wires that the mutual inductance coefficient is difficult to determine directly. To simplify the theoretical calculation, according to this layer, the maximum value of the total inductance generated by the mutual inductance of the wires is calculated, that is, the mutual inductance coefficient between the wires is the maximum value of one. At this time, the total mutual inductance of one wire is the sum of the inductances of multiple wires and the total mutual inductance received by multiple wires; that is, it is the product of the square of the inductance of a single wire and the number of wires. By denoting *x* as the number of wires’ roots, the corresponding formula can be expressed by

(11) $$\begin{array}{c} L_{layer}=x^{2}L_{line} \end{array}$$

#### 2.2.3. A Model for Calculating the Coil Capacitance

The capacitance value of the coil refers to the parasitic capacitance that appears after the coil is made. Generally speaking, the parasitic capacitance appears in two main factors: the PCB circuit board between the layers of the aperture capacitance and the capacitance generated between the wires. Herein, the parasitic capacitance is essentially generated by the capacitance between the layers of the circuit board and the layers of the positive conductor.

The primary and secondary coils can be approximated as a parallel plate capacitor. The area directly opposite the two is the area of the secondary coil. Since the plate represents two pairs of coils, the calculated interlayer parasitic capacitance multiplied by two can be regarded as the size of the parasitic capacitance of the whole plate. Therefore, based on the parallel plate capacitance formula, the coil parasitic capacitance is given by

(12) $$\begin{array}{c} C_{coil}=2\left(\frac{\varepsilon_{0}\varepsilon_{r}S}{h_{B}}\right) \end{array}$$

where $\varepsilon_{0}$ represents the vacuum dielectric constant with a value of 8.85 × 10^−12^ F/m,$\;\varepsilon_{r}$ denotes the relative permittivity, *S* is the square area, and $h_{B}$ denotes the distance between the plates.

### 2.3. PCB Design

The elimination of inductive effects in the present work is essentially based on the extremely high degree of coupling of the two coils. To achieve this goal, a high degree of regularity of the coil shape and positive angularity is required, which is difficult to achieve with ordinary coils wound through enameled wires. According to the design requirements, printed circuit board coils (PCB coils) are utilized to ensure the regularity of the coils.

Obviously, the PCBs used as coils need to have the following characteristics, which can be met by conventional FR-4 materials. The thicker and wider the copper foil, the lower the resistance value, but increased thickness and width could lead to poor adhesion between the copper foil and the circuit board substrate. This could result in the peeling of the copper foil from the board, and the circuit board being layered layer by layer. In other words, the closer the thickness of the substrate, the better the coupling effect, but the thinner the thickness of the substrate, the lower the mechanical strength of the PCB. Therefore, these parameters should be integrated into the design requirements and practical process constraints to determine the parameters.

After cooperation and consultation with PCB manufacturers, the PCB processing parameters are presented in Table 2.

### 2.4. Design and Evaluation of the Coil Parameters

In the present design, the set of PCB processing parameters is presented in Table 2.

When creating PCBs to provide high-power outputs, it is necessary to first perform the relevant safety checks on the board. The main consideration for the circuit board is whether the temperature rise of the PCB exceeds the safe temperature of the materials used in the PCB at the design limits, resulting in damage.

Considering that the coil wire designed in this paper passes a high-frequency current, it is inappropriate to use the general steady-state circuit current safety calibration. It is only necessary to consider that the heating generated by the wire during the passage time of the high-frequency current would be in the safe range, so the short-circuit allowable current formula was employed to evaluate the limiting current.

Yang [13], in estimating the PCB circuit temperature, gave the equation for the allowable current of the PCB copper foil in a short-circuit condition as follows:

(13) $$\begin{array}{c} I_{MAX}=152wh_{L}\sqrt{f_{MAX}} \end{array}$$

Further calculations yield a maximum limiting current of 880.87 A for the PCB copper foil.

### 2.5. Coil Shape Design

Currently, common PCB coils are round, square, and other square polygons. According to an investigation conducted by Zhu [14] on planar spiral inductors using polygons, the number of sides of a square polygon is directly proportional to the inductance while keeping the same inner and outer diameters. In particular, the inductance of a circular spiral coil with a diameter of 200 mm is 1.24 times greater than that of a square coil with a side length of 200 mm when all other factors are considered the same [14].

However, the exploitation of square coils still possesses major drawbacks. Yang [13], in analyzing the PCB alignment on the interference of high-frequency signals, explicitly suggested that right-angle routing on printed circuit boards plays a negative role in the formation of high-frequency signals. The corner may be equivalent to a capacitive load on the transmission line, which pulls up the rise time to the detriment of high-frequency signal formation, and the right-angled tip generates additional electromagnetic interference (EMI). Therefore, it is necessary to minimize the presence of right angles in the coil shape.

Combining the findings of both, the shape of the coil was designed as a runway-type coil with a square body and generally rounded corners. A suitable combination of two conventional coils avoids the shortcomings of both, reduces inductance and external interference in appearance, and minimizes impediments to high-frequency, high-current signal generation.

**Figure 5:**
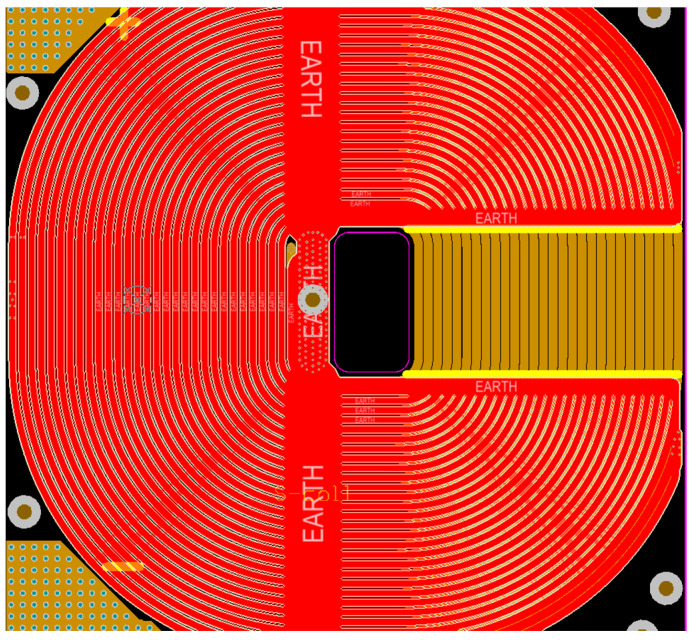
Design of the secondary coil covering the main coil.

The coil employs a helical track-type coil to connect a high-frequency circuit for current transmission, called the primary coil. As illustrated in Figure 5, the other two layers are printed with corresponding coils to counteract the inductance of the coil, called secondary coils. As can be seen in Figure 5, there exists a portion of the main coil that has not yet been masked, and this portion is the test window where the coil is available for outside testing.

## 3. Materials and Parameters

**Figure 6:**
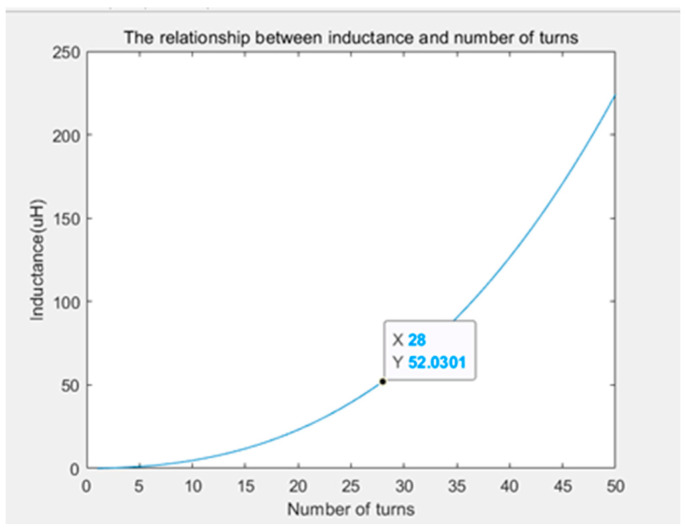
Plot of the inductance in terms of the number of turns.

After completing the work related to coil design, the coil parameters should be appropriately designed. For this purpose, the coil is approximated as a circular helical coil, according to the reference value given by Zhao [11]. Let us set the values of C1, C2, C3, and C4 equal to 1, 2.46, 0, and 0.2, respectively. Take the number of turns as the independent variable and inductance as the dependent variable, and then bring them into the formula for calculation to obtain the graph of Figure 6 (i.e., demonstrating the relationship between the number of turns and inductance).

It can be seen that the inductance exponentially rises with the number of turns, so from the point of view of reducing inductance, the fewer the number of turns the better. Nevertheless, the fewer the number of turns, the greater the burden on a single wire when the output reaches the design amplitude current.

In engineering applications, when the value of the function cannot be too large or too small in actual use, the closest value to 1 can be considered. The point corresponding to the slope of the tangent line of the function is used as the appropriate value. A tangent point with a slope of 1 represents the degree of change of the dependent variable and the independent variable of the function at this time, which can avoid the situation of the value being too large or too small [9]. In this case, the value of the point is 28, which means that the number of turns of the single-turn coil selection is 28 turns. At this time, one can write

(14) $$\begin{array}{c} I_{Singlecoil-MAX}=3\times 10^{4\;}A/28\approx 1071.42\;A>I_{MAX}=880.87\;A \end{array}$$

This coil carries a current at the extreme index of $I_{Singlecoil-MAX}$ that far exceeds $I_{MAX}$, which is clearly unsuitable.

Let us improve the coil by connecting two pairs of primary and secondary coils in series on a PCB board. For this purpose, a 4-layer PCB board was utilized and the number of turns of the coil on a single board was increased to 56 turns. Therefore,

(15) $$\begin{array}{c} I_{Twocoil-MAX}=3\times 10^{4}\;A/56\approx 535.71\;A<I_{MAX} \end{array}$$

At this point, it can be assumed that the PCB coil could work safely and normally in the presence of the limit index.

**Figure 7:**
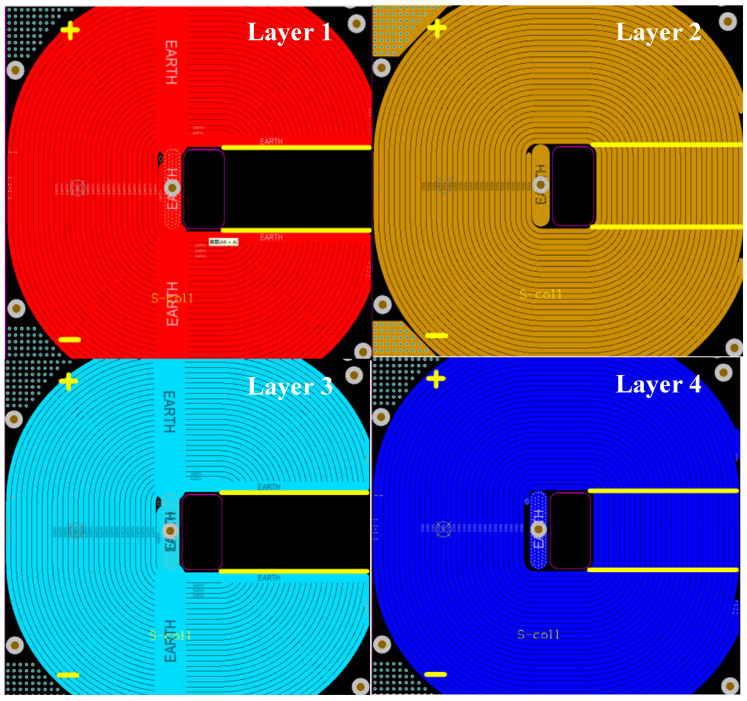
Design of each layer of 4-layer PCB coil.

To minimize unwanted leakage and the loss of magnetic energy, an additional coil of wire was added to the outside of the coil. A grounding layer was also involved in the coil bore located in front of the test window to provide electromagnetic shielding. These new strategies complete the overall design, as presented in Figure 7.

After completing the design and measuring some factors, we can obtain the dimensional parameters that should be brought into the calculation (see Table 3).

## 4. Results

**Figure 8:**
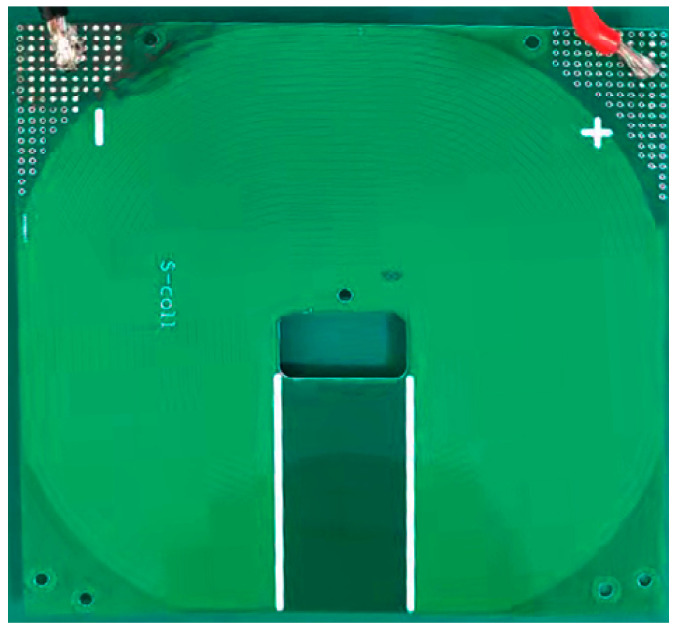
Physical image of the non-inductive coil.

**Figure 9:**
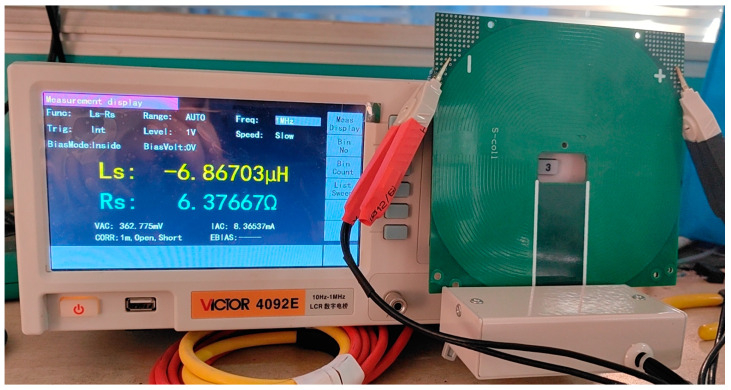
Inductance and impedance test setup of the LCR bridge.

After the structural design and theoretical verification were completed, the physical object of this coil was fabricated as illustrated in Figure 8:

The inductance and impedance of the PCB coil were measured directly using the LCR bridge, the model is VICIOR 4092E, under a sine wave at a frequency of 1 MHz, as demonstrated in Figure 9.

It should be noted that due to parasitic capacitance and other parameters that affect the measurement of passive components, the inductance measurement may have negative values, which indicates that the inductance of the object being measured is much smaller than its own capacitance, and the capacitance characteristics of the object being measured are higher than the inductance characteristics.

As can be seen in Figure 9, the inductance value is −6.86 μH. A spiral coil shows capacitance characteristics in the bridge measurement. Such a fact proves that the inductance of this coil is much smaller than the parasitic capacitance of the coil itself, and the parasitic capacitance does not influence the formation and output of high-frequency currents. This issue is in line with the article on reducing the effect of inductance, and the design goal is to hinder the resistance of high-frequency and large currents. Additionally, the measured impedance is approximately 6.37 Ω, which is similar to the results of the theoretical calculations, verifying the validity and reference value of the theoretical calculations.

**Figure 10:**
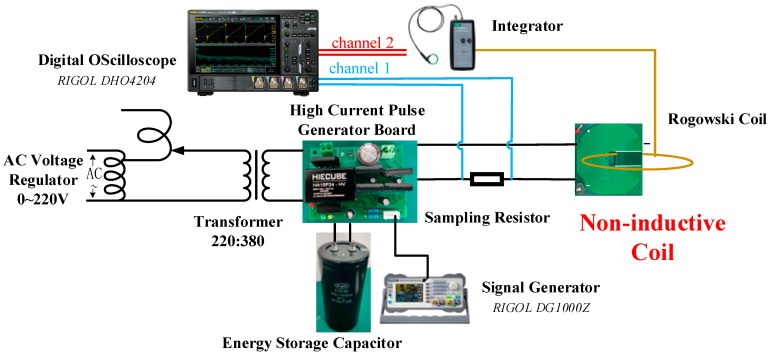
Structure of the test platform building.

To verify whether the actual use of the non-inductive coil meets the design specifications, a current source test platform was suitably constructed for the output coil, and the structure of the platform is schematically presented in Figure 10.

The test platform utilizes an AC voltage regulator to increase the 220 V AC input to 380 V AC, which is supplied to the storage capacitor for charging. It employs the signal generator to control the high-frequency current parameters through the IGBT control capacitor voltage at both ends according to the signal waveform released on the coil. The current is multiplied through the coil to obtain a high-frequency current.

**Figure 11:**
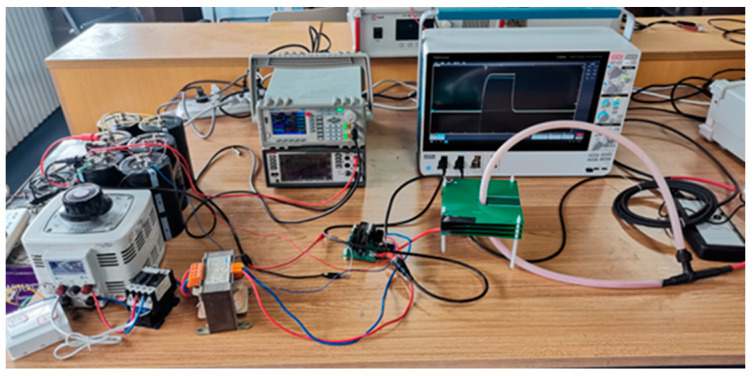
Actual construction of the test platform.

The single-turn current output from the test platform is detected by shunt sampling, and the waveforms sampled from the shunt can be regarded as the real waveform output from this current source. At the same time, to check the quality of the high-frequency current output from the current source, the output current of the current source is captured at the output power coil end using a Roche coil, which is altered to a voltage signal through an integrator to be shown on an oscilloscope. The actual construction of the above-mentioned platform is illustrated in Figure 11.

**Figure 12:**
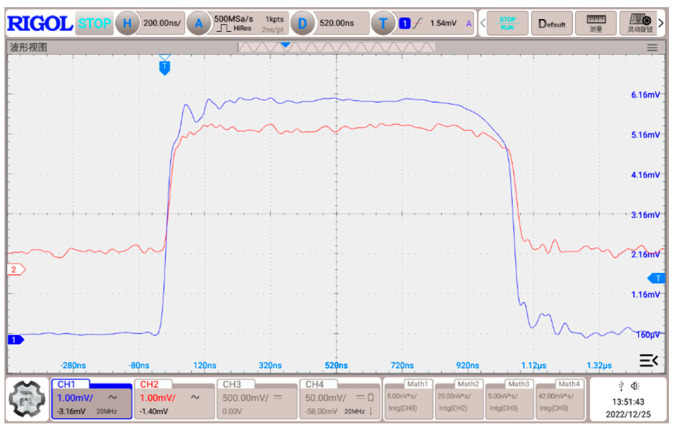
Test waves.

After the construction is completed, a 1 MHz single-cycle square wave is generated by the signal generator, the pulse is output through the test platform and the test coil, and the pulse waveform is sampled through the shunt after 1000 times of attenuation. The test results are illustrated in Figure 12.

The blue curve of channel 1 of this oscilloscope in Figure 12 shows the current waveform of the voltage signal measured by the shunt, and the red curve of channel 2 illustrates the voltage signal measured by the Roche coil from the output.

The low value of the voltage waveform before the rising edge represents the zero-input state of the system when no current flows through it. When the current pulse appears, the waveform exhibits a rising edge. The shorter the rise time, the smaller the parasitic inductance of the output coil; after that, the waveform reaches stability. The difference between the high value and the low value represents the amplitude, which signifies the amplitude of the current pulse flowing through the sampling resistor.

From Figure 12, it can be seen that for the 1 MHz high-frequency square wave, its high-frequency rising edge is about 20 ns. While comparing the sampled current source signal and the Roche coil at the output end of the measured signal, it is detectable that the two signals have the same waveform trend, the waveform rising time delay is not obvious, and the waveform variation along the waveform platform does not exhibit a noticeable slow slope.

The shape of the waveform and the rising edge of the test results demonstrate that the output of the inductive reactance of the non-inductive coil is low, the inductive reactance for the generation of high-frequency signal obstruction effect is not obvious, the output of the high-frequency, high-current pulse waveform fidelity is good, and no obvious distortion loss of waveform information is detected.

Let us calculate the current output amplitude for Figure 12. The current amplitude calculation for this test rig can be formulated as

(16) $$\begin{array}{c} I_{OUTPUT}=\frac{U_{amplitude}}{R_{Sample}}\times 56\times 1000 \end{array}$$

where *U_amplitude_* represents the amplitude of the high-frequency current value, and, according to Figure 12, it can be read in the amplitude value of about 6.02 mV. In addition,$\;R_{Sample}$ denotes the value of the sampling resistance, namely 1.091 mΩ; 56 is the number of turns of the coil; and 1000 is the attenuation of the sampling resistance relative to a single-turn coil time. According to the measurement results in Figure 12, the voltage amplitude value collected by the sampling resistor is about 6.16 mV, and the current amplitude value calculated according to Equation (16) is about 33 kA.

We summarize the main parameters of the waveforms measured by the sampling resistor and the Rogowski coil in Figure 12 and Table 4.

**Figure 13:**
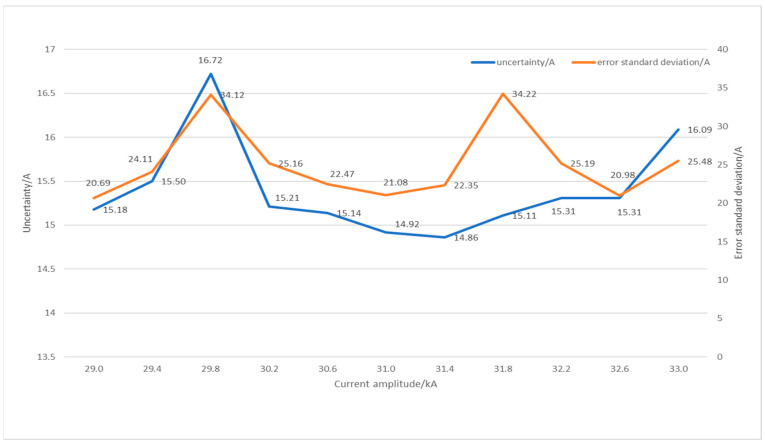
Test results.

Since the high-precision sampling resistor is connected to the output port of the pulse current source and the current enters the PCB coil after passing through the sampling resistor, the measured amplitude parameter can be considered the true value of the output current amplitude of the current source. The current waveform measured by the Rogowski coil is regarded as the measured value of the coil output current. The absolute value of the difference between the two can be taken as the uncertainty of the coil. We adjusted the current source output so that the output current range was between 33 kA and 29 kA, and then we repeated the experiment 400 times, with 0.4 kA as the one grid, averaged the measured uncertainty into 10 characteristic points, and created the uncertainty table in depicted Figure 13.

The plotted results in Figure 13 reveal that the maximum output uncertainty of the coil is 16.72 A, and the maximum standard deviation is 34.12 A. For the design range of 30 kA, the output error range at various amplitudes is less than 0.06%, and the output stability error range is less than 0.12%. The coil has robustness with various output ranges and repeatability.

According to Table 1, the output coil specifications meet the rising edge within 25 ns, an output current amplitude of 30 kA, and an output waveform standard 1 Mhz square wave.

## 5. Comparison of Theoretical Calculation Effects

Based on theoretical simulations, the difference in inductance values between the coil without the new structural design and the coil with the structural design is discussed, whereas other parameters are the same. Suppose that the design of transformer mutual coupling is not adopted and the main coil is directly connected to the circuit. In this case, first, the inductance of the single-layer coil of the main coil is calculated.

Referring to Equation (8) and Table 3, the inductance of a single-layer coil (*L_layer_*) is evaluated to be about 64 μH.

At this time, we also should consider the two layers of the main coil in the same PCB, which will inevitably produce mutual inductance between the coils. As the two coils of the current flow in the same direction, the coil mutual inductance generated by the inductance and the self-inductance generated by the same direction of the induced voltage, together with the high-frequency circuit, play a crucial role in hindering the need to calculate the inductance of the mutual inductance.

According to Equation (4), the main coil of the two layers is completely consistent, and the self-inductance is consistent. The formula can be simplified as $L_{M}$ *= kL*, where *k* signifies the coupling coefficient.

For the inductance between PCB coils, Jonsener Zhao [11] provided an engineering estimation formula for k for the mutual inductance between multiple layers of PCB spiral coils with less than 20 turns. According to the literature, this formula still possesses reference value as an approximate calculation when the outer coil diameter is much greater than the distance between the two coils’ layers in the case of the number of turns exceeding 20 [4,7].

The engineering estimation formula for *k* based on the work of Zhao [11] is as follows:

(17) $$\begin{array}{c} k=\frac{N^{2}}{0.64\left[\left(0.184X^{3}\;-\;0.525X^{2}\;+\;1.038X+1.001\right)\left(1.67N^{2}\;-\;5.84N\;+\;65\right)\right]}, \end{array}$$

where *N* is the number of coil turns and *X* represents the distance between two layers. According to the design, the distance between the two main coils is set to 1.54 mm. The calculated value of *k* is approximately obtained as 0.9950.

The total inductance of the two-layer coil can be evaluated as follows:

(18) $$\begin{array}{c} L_{SUM-coil}=L_{1}+L_{2}+L_{M}=\left(2+k\right)L \end{array}$$

where the calculation result leads to 191.68 μH.

As Equation (10), this can be viewed as the inductance generated by a straight wire, which is brought into the calculation to give a total inductance of 1528.8 nH.

Let us calculate the inductance of a board coil based on Equation (15), which is constituted of one board and two layers. By doing so, the calculated value is obtained as 4.58 μH.

As can be seen, by comparing the above value with 191.68 μH, the inductance is reduced by about 42 times, indicating that this design could substantially reduce the coil inductance.

From Equation (7), the resistance of the single-layer coil at this point equals approximately 2.13 Ω. Due to the similarity of the two coils under consideration, the primary coil itself has a resistance of 2.13 Ω. In contrast to the ideal secondary coil, this secondary coil does not exhibit the resistance of the test window and has the additional resistance of the wires connecting the coil circuitry. Comparing the wire lengths of layer 1 and layer 2, due to the presence of a test gap in layer 1, layer 1 has a 0.77 m shorter wire length than layer 2. Calculations show that the resistance of this part is only 0.21 Ω, resulting in an AC impedance of 4.05 Ω. The impedance of the double-layer circuit board is only 8.10 Ω.

The parasitic capacitance and impedance of the coil are also given here in a simple calculation. By substituting the value from Table 2 into Equation (12), the value of parasitic capacitance is calculated as 1.74 pF. This capacitance value is much smaller in magnitude than the coil resistance and coil inductance and can be approximated as an insulating state. As a result, the effect of parasitic capacitance can be rationally ignored.

According to Figure 11, the theoretically calculated inductance is obtained as 4.58 μH, which is in the same order of magnitude as the measured 6.69 μH. Additionally, the theoretically calculated impedance is 8.10 Ω, which is in the same order of magnitude as the measured 6.96 Ω. This fact reveals that the theoretical calculation is close to the actual measurement result, and the theoretical calculation has a reference significance. This fact explains that the theoretical calculation is close to the actual measurement results, and the theoretical calculations are of reference significance.

## 6. Conclusions

This paper accomplishes the design of a non-inductive coil for high-frequency current output. The output current method based on the ampere-turn method is designed and the principle of coil inductance generation is discussed. The structures of the main and secondary coils are suitably designed on the basis of the principle of inductance and the law of conservation of energy to eliminate the effect of inductance.

After completing the establishment of the theoretical model, the shape and dimensional pa-rameters of the coil were determined according to the design requirements, and the key electromagnetic parameters of the coil was calculated. From the theoretical calculation, it can be concluded that the structure of the non-inductive coil is reasonable, and the inductance is 42 times smaller than that of the coil without eliminating the effect of inductance, with the value reduced from 191.68 μH to 4.58 μH. According to the physical verification, the actual inductance of this coil is predicted to be 6.69 μH after the measurement by the LCR bridge, and the coil is capable of outputting 1 MHz with an amplitude of 33 kA. After connecting to a high-frequency current source, the coil can output a current pulse of 30 kA with a rise time of 20 ns and an amplitude of 30 kA. Additionally, since the influence of inductance on the high-frequency current is eliminated, the generated high-frequency current signal exhibits good smoothness and recovery and is convenient for equipment diagnosis.

## 7. Future Works

It can be seen that the theoretically calculated electromagnetic parameters of the output coil are in good agreement with the physical measurements. However, the design is limited by the fact that the instantaneous current carried by the PCB copper foil hardly exceeded 1 kA, so the output amplitude is much lower than that of the leading current source. In fact, there is still a lack of sufficient electromagnetic explanation for the parasitic parameters of the coil and the nature of the capacitive properties at high frequencies. This part of the work still requires further investigation in the future.

## Figures and Tables

**Table 1:** High-frequency current source output limits design requirements.

Norm	Parameters
The amplitude current (*I_MAX_*)	30 kA
Maximum frequency (*f_MAX_*)	1 MHz
Rise time	25 ns

**Table 2:** The PCB processing parameters.

Processing Indicator	Parameter
PCB sheet	FR-4
Relative dielectric constant of substrate $\varepsilon_{r}$	1 MHz
Copper resistivity	1.75 × 10^−8^ Ω·m
Conductor line width (*w*)	1.85 mm
Thickness of conductor (*h_L_*)	0.035 mm
Distance between plates (*h_B_*)	0.0254 mm

**Table 3:** Main parameters pertinent to the coil design.

Design Parameter	Value
Outer diameter (*D*)	145.5 mm
Internal diameter (*d*)	33.8 mm
Spacing (*h*) Number of turns (*N*)	0.1 mm 28 × 2
Coil wire length ($l_{length}$)	9.7 m
Distance between two layers of coils (*H*)	1.5 mm
Coil facing area (*S*)	1.22 × 10^−4^ m^2^

**Table 4:** Main waveform parameters.

Wave Parameters	Sampling Resistance Measurement	Rogowski Coil Measurements
Voltage amplitude (mV)	5.89	2.92
Rise time (ns)	20	20
Current amplitude (kA)	32.984	32.976

## Data Availability

Data are contained within the article.
